# Supplementary figures and images for: Bacteriophage activity against and characterisation of avian pathogenic Escherichia coli isolated from colibacillosis cases in Uganda
Source: PLoS One. 2020 Dec 15;15(12):e0239107. doi: 10.1371/journal.pone.0239107 (PMC7737885; doi:10.1371/journal.pone.0239107)

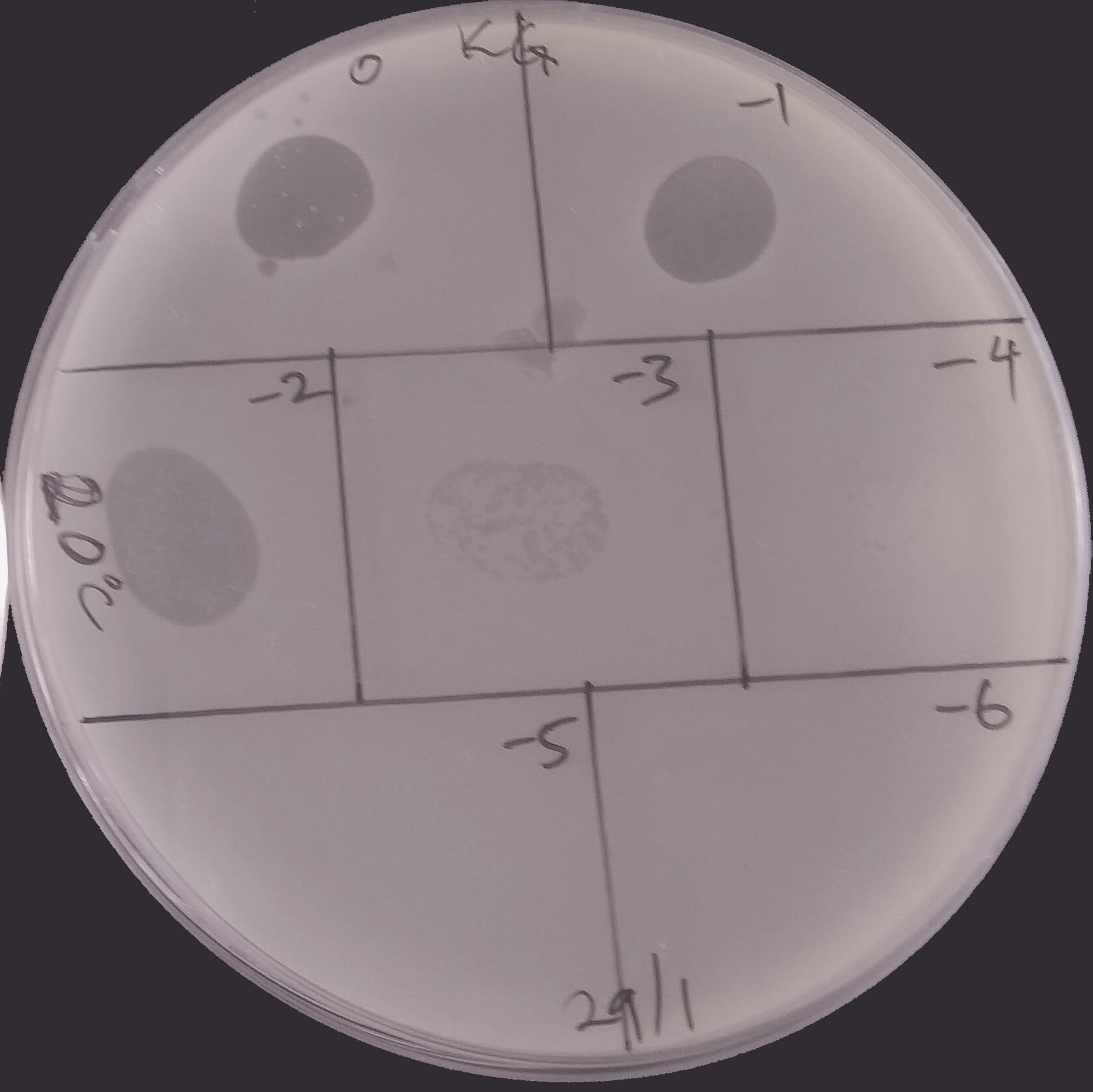

Supplement: S1 Fig — The grid lines indicate the different dilutions of the phage suspension. Plaque assay was carried out by the spot assay method. (TIF) [file pone.0239107.s001.tif]
