# Supplementary material for: Bacteriophage activity against and characterisation of avian pathogenic Escherichia coli isolated from colibacillosis cases in Uganda
Source: PLoS One. 2020 Dec 15;15(12):e0239107. doi: 10.1371/journal.pone.0239107 (PMC7737885; doi:10.1371/journal.pone.0239107)
Supplement: S1 Table — Sensitivity patterns of the seven phages on the 14 APEC isolates. (DOCX) [file pone.0239107.s002.docx]

**S1 Table. Host Range - Sensitivity patterns of the seven phages on the 14 APEC isolates**

| **APEC**  **Isolate ID** | **Phage isolate ID** | | | | | | |  |
| --- | --- | --- | --- | --- | --- | --- | --- | --- |
|  | **UPEC01** | **UPEC03** | **UPEC04** | **UPEC06** | **UPEC08** | **UPEC09** | **UPEC10** | **No. of**  **phages**  **active**  **against**  **the APEC** |
| 23 | - | - | + | - | - | - | - | 1 |
| S3Lug | + | + | - | + | + | - | + | 5 |
| 20 | - | + | + | + | - | - | + | 4 |
| S1bC13 | - | - | - | - | - | - | + | 1 |
| C8S1 | - | - | - | - | - | - | + | 1 |
| 2 | + | + | + | + | + | + | + | 7 |
| S3SPEC | - | - | + | - | - | - | - | 1 |
| 28/1792 | - | - | + | - | - | - | - | 1 |
| C6S14 | - | - | + | - | - | - | - | 1 |
| 19-10952 | - | - | + | - | - | - | - | 1 |
| 19-1330 | - | - | + | - | + | - | - | 2 |
| 19-1331 | + | + | + | + | - | - | - | 4 |
| 19-10951 | + | + | + | + | + | - | - | 5 |
| S2Ecoli2 | - | - | - | + | - | - | + | 2 |
| **No. of APEC isolates lysed** | **4** | **5** | **10** | **6** | **4** | **1** | **6** |  |

**+, Lysis; - , No lysis**
