# Supplementary material for: Bacteriophage activity against and characterisation of avian pathogenic Escherichia coli isolated from colibacillosis cases in Uganda
Source: PLoS One. 2020 Dec 15;15(12):e0239107. doi: 10.1371/journal.pone.0239107 (PMC7737885; doi:10.1371/journal.pone.0239107)
Supplement: S1 File — (DOCX) [file pone.0239107.s003.docx]

**S1 File. PCR method for detecting the virulence genes**

Amplification of the virulence genes was carried out following a method described by Johnson *et al* (2008). Briefly, the reaction mixture consisted of; 5μL of 5× MyTaq Red Reaction Buffer (BIOLINE), 0.75μL of each primer (Eurofins), and 2U MyTaq Red DNA polymerase (BIOLINE), making up a total volume of 25μL of the reaction mixture. The PCR reactions were performed under the following parameters; 94°C for 2 min; 25 cycles of 94°C for 30 s, 63°C for 30 s, 68°C for 3 min; and a final cycle of 72°C for 10 min. The PCR products were analyzed by horizontal gel electrophoresis in 4% agarose. The primer sequence for the selected virulence genes are presented in Table S2. An isolate was considered to contain a gene of interest if it produced an amplicon of the expected size.

**Table S2. Primer sequences and the amplicon sizes of the selected *E. coli* virulence genes (sourced from Johnson *et al*., (2008).**

| **Gene** | **Primer Sequence (5’ to 3’)** | **Amplicon size (bp)** |
| --- | --- | --- |
| *iroN* | F: AATCCGGCAAAGAGACGAACCGCCT  R: GTTCGGGCAACCCCTGCTTTGACTTT | 553 |
| *ompT* | F: TCATCCCGGAAGCCTCCCTCACTACTAT  R: TAGCGTTTGCTGCACTGGCTTCTGATAC | 496 |
| *hlyF* | F: GGCCACAGTCGTTTAGGGTGCTTACC  R: GGCGGTTTAGGCATTCCGATACTCAG | 450 |
| *iss* | F: CAGCAACCCGAACCACTTGATG  R: AGCATTGCCAGAGCGGCAGAA | 323 |
| *iutA* | F: GGCTGGACATCATGGGAACTGG  R: CGTCGGGAACGGGTAGAATCG | 302 |
