# Supplementary material for: Bacteriophage activity against and characterisation of avian pathogenic Escherichia coli isolated from colibacillosis cases in Uganda
Source: PLoS One. 2020 Dec 15;15(12):e0239107. doi: 10.1371/journal.pone.0239107 (PMC7737885; doi:10.1371/journal.pone.0239107)
Supplement: S2 File — (DOCX) [file pone.0239107.s004.docx]

**S2 File. PCR method for determining phylogenetic groups**

Multiplex PCR followed a method described by Clermont *et al* (2000). Briefly, PCR was carried out in a 25μL volumes containing 5 μL of 5× MyTaq Red Reaction Buffer, 2 μL of each primer, 2U of MyTaq Red DNA polymerase (BIOLINE) and 1 μL of template DNA. The PCR conditions were as follows: denaturation for 5 min at 94°C; 30 cycles of 30 s at 94°C, 30 s at 55°C, and 30 s at 72°C; and a final extension step of 7 min at 72°C. The PCR products were visualized under ultraviolet light after electrophoresis on a 2% agarose gel. Categorization as phlyogenetic group A, B1, B2, and D depended on detection of the *YjaA*, TspE4.C2 and *ChuA* genes or their combination as described by Clermont *et al* (2000). Details of primers used are listed in Table S3.

**Table S3. Primer sequences and their amplicon sizes for the determination of *E. coli* phylogenetic groups (sourced from Clermont *et al*, (2000)**

| **Target Gene** | **Primer sequence (5’ to 3’)** | **Amplicon size (bp)** |
| --- | --- | --- |
| *ChuA* | F: GACGAACCAACGGTCAGGAT  R: TGCCGCCAGTACCAAAGACA | 279 |
| *yjaA* | F: TGAAGTGTCAGGAGACGCTG  R: ATGGAGAATGCGTTCCTCAAC | 211 |
| *Tsp*E4.C2 | F: GAGTAATGTCGGGGCATTCA  R: CGCGCCAACAAAGTATTACG | 152 |
