# Supplementary material for: Bacteriophage activity against and characterisation of avian pathogenic Escherichia coli isolated from colibacillosis cases in Uganda
Source: PLoS One. 2020 Dec 15;15(12):e0239107. doi: 10.1371/journal.pone.0239107 (PMC7737885; doi:10.1371/journal.pone.0239107)
Supplement: S3 File — (DOCX) [file pone.0239107.s005.docx]

**S3 File. PCR method for detection of APEC serotypes**

PCR was carried out following a method described by Wang *et al* (2014) as follows; The PCR reaction mixture (25μL) was composed of; 1 μL template DNA; 5μL of 5× MyTaq Red Reaction Buffer (BIOLINE), 2U MyTaq Red DNA polymerase (BIOLINE), and 0.5 μL (10 μM) of each primer pair. The PCR conditions included denaturation at 95℃ for 5 min, followed by 30 cycles of 95℃ for 35 s, 57℃ for 30 s, 72℃ for 40 s and a final extension at 72℃ for 10 min. The PCR products were analyzed under ultraviolet light after electrophoresis on a 2% agarose gel. Details of the primer sequences are listed in Table S4.

**Table S4. Primer sequences and their amplicon sizes for the detection of O1, O2, and O78 *E. coli* serogroups (sourced from Wang *et al*. 2014)**

| **Target gene** | **Primer sequence (5’ to 3’)** | **Amplicon size (bp)** |
| --- | --- | --- |
| *rfb* O1 | F: CGATGTTGAGCGCAAGGTTG R: CATTAGGTGTCTCTGGCACG | 263 |
| *rfb* O2 | F: CGATGTTGAGCGCAAGGTTG R: GATAAGGAATGCACATCGCC | 355 |
| *rfb* O78 | F: CGATGTTGAGCGCAAGGTTG R: TAGGTATTCCTGTTGCGGAG | 623 |
