# Supplementary material for: Bacteriophage activity against and characterisation of avian pathogenic Escherichia coli isolated from colibacillosis cases in Uganda
Source: PLoS One. 2020 Dec 15;15(12):e0239107. doi: 10.1371/journal.pone.0239107 (PMC7737885; doi:10.1371/journal.pone.0239107)
Supplement: S1 Results — (DOCX) [file pone.0239107.s006.docx]

**Table showing presence of the five virulence genes (*iutA, iss, hlyF, ompT*, and *iroN*) of APEC**

| **S/No** | **Sample ID** | ***iutA*(302bp)** | ***iss*(323bp)** | ***hlyF*(450bp)** | ***ompT*(496bp)** | ***iroN*(553bp)** | **Total** |
| --- | --- | --- | --- | --- | --- | --- | --- |
| 1 | AP3 | 1 | 1 | 1 | 1 | 1 | 5 |
| 2 | S1Lung | 1 | 1 | 1 | 1 | 1 | 5 |
| 3 | 176 | 1 | 1 | 1 | 1 | 1 | 5 |
| 4 | S2Lung | 1 | 0 | 1 | 1 | 0 | 3 |
| 5 | 106 | 1 | 1 | 1 | 1 | 1 | 5 |
| 6 | 105 | 1 | 1 | 1 | 1 | 1 | 5 |
| 7 | 171/25/02 | 1 | 1 | 1 | 1 | 1 | 5 |
| 8 | C24S11 | 1 | 0 | 0 | 0 | 0 | 1 |
| 9 | AP2 | 1 | 1 | 1 | 1 | 1 | 5 |
| 10 | 53 | 0 | 0 | 1 | 1 | 0 | 2 |
| 11 | 109 | 1 | 1 | 1 | 1 | 1 | 5 |
| 12 | S2Ecoli | 0 | 1 | 0 | 1 | 1 | 3 |
| 13 | C28S1 | 0 | 1 | 0 | 1 | 1 | 3 |
| 14 | S29C12 | 1 | 1 | 1 | 1 | 1 | 5 |
| 15 | S2EC | 1 | 1 | 1 | 1 | 1 | 5 |
| 16 | C7S14 | 1 | 1 | 1 | 1 | 1 | 5 |
| 17 | 137/28/02 | 1 | 1 | 1 | 1 | 1 | 5 |
| 18 | C8S3 | 0 | 1 | 1 | 1 | 1 | 4 |
| 19 | C29SIN | 1 | 0 | 0 | 0 | 0 | 1 |
| 20 | S29S23 | 1 | 1 | 1 | 1 | 1 | 5 |
| 21 | 26 | 1 | 1 | 1 | 1 | 0 | 4 |
| 22 | C24S29 | 1 | 1 | 1 | 1 | 0 | 4 |
| 23 | EcoliS3 | 1 | 0 | 0 | 0 | 0 | 1 |
| 24 | C24S1b | 1 | 1 | 1 | 1 | 1 | 5 |
| 25 | C14S29 | 0 | 1 | 0 | 0 | 0 | 1 |
| 26 | C24S3b | 1 | 1 | 1 | 1 | 0 | 4 |
| 27 | S44C24 | 1 | 1 | 1 | 1 | 0 | 4 |
| 28 | 78/28/02 | 0 | 0 | 1 | 1 | 0 | 2 |
| 29 | C9S3 | 1 | 1 | 1 | 1 | 0 | 4 |
| 30 | S19C25 | 1 | 0 | 0 | 0 | 0 | 1 |
| 31 | C29 | 1 | 1 | 1 | 1 | 1 | 5 |
| 32 | S2Ecoli2 | 1 | 1 | 1 | 1 | 1 | 5 |
| 33 | C8S19 | 1 | 1 | 1 | 1 | 0 | 4 |
| 34 | C9S19 | 1 | 1 | 1 | 1 | 0 | 4 |
| 35 | S3C28 | 1 | 1 | 1 | 1 | 1 | 5 |
| 36 | S3HEC | 1 | 1 | 1 | 1 | 1 | 5 |
| 37 | 19-1330 | 1 | 1 | 1 | 1 | 1 | 5 |
| 38 | 19-10952 | 0 | 1 | 1 | 1 | 1 | 4 |
| 39 | S14 | 1 | 0 | 0 | 0 | 0 | 1 |
| 40 | Ecoli | 0 | 0 | 0 | 0 | 0 | 0 |
| 41 | 3PC | 0 | 0 | 0 | 0 | 0 | 0 |
| 42 | C24S3b(2) | 0 | 0 | 0 | 0 | 0 | 0 |
| 43 | 8 | 0 | 0 | 0 | 0 | 0 | 0 |
| 44 | C13S1b | 0 | 0 | 0 | 0 | 0 | 0 |
| 45 | 28/179/02 | 0 | 0 | 0 | 0 | 0 | 0 |
| 46 | C10S19 | 0 | 0 | 0 | 0 | 0 | 0 |
| 47 | 19 | 0 | 0 | 0 | 0 | 0 | 0 |
| 48 | 2 | 0 | 0 | 0 | 0 | 0 | 0 |
| 49 | APEC | 0 | 0 | 0 | 0 | 0 | 0 |
| 50 | 23 | 0 | 0 | 0 | 0 | 0 | 0 |
| 51 | 142 | 0 | 0 | 0 | 0 | 0 | 0 |
| 52 | 20 | 0 | 0 | 0 | 0 | 0 | 0 |
| 53 | 8 | 0 | 0 | 0 | 0 | 0 | 0 |
| 54 | C8S1 | 0 | 0 | 0 | 0 | 0 | 0 |
| 55 | S3Lug | 0 | 0 | 0 | 0 | 0 | 0 |
| 56 | 19-10951 | 0 | 0 | 0 | 0 | 0 | 0 |
| 57 | 19-1331 | 0 | 0 | 0 | 0 | 0 | 0 |
| 58 | 135 | 0 | 0 | 0 | 0 | 0 | 0 |
|  | **Total** | **32** | **31** | **31** | **33** | **23** | **150** |

1= Present, 0 = Absent

**Agarose gels showing the virulence gene profiles of APEC from a multiplex PCR**

**Virulence genes and their band sizes**

| **Gene** | **Band size in bp** |
| --- | --- |
| *iutA* | 302 |
| *Iss* | 323 |
| *hlyF* | 450 |
| *ompT* | 496 |
| *iroN* | 553 |


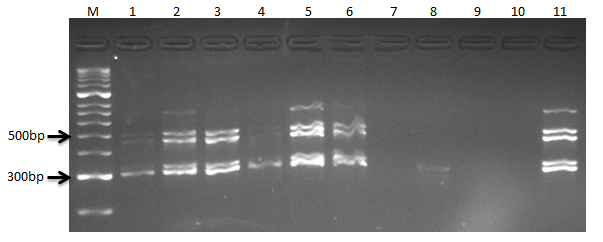
Samples (1A-11A) 6.7.2019


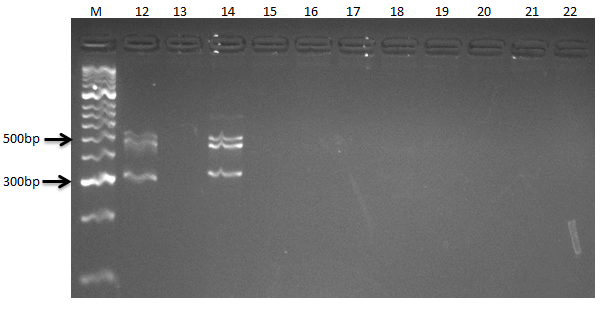


Samples (12A-22A) 6.7.2019


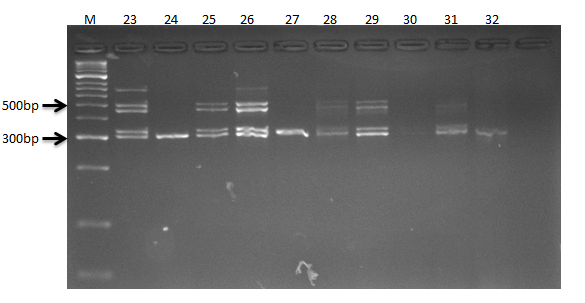
Samples (91-910) 15.7.2019

Samples (911-920) 15.7.2019
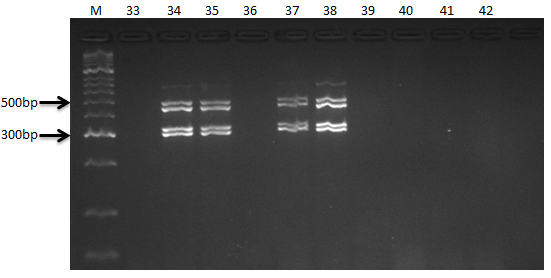


Samples (101-1010) 18.7.2019


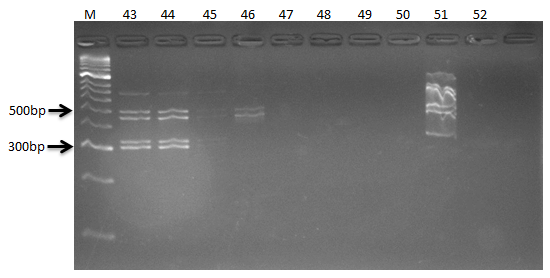


Samples (1011-1020) 18.7.2019


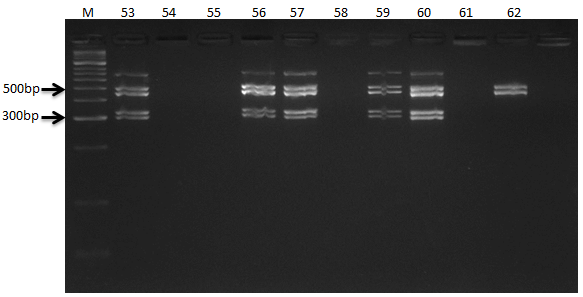


| **Lane Number** | **Sample ID** |
| --- | --- |
| M | Marker |
| 1 | AP3 |
| 2 | S1Lung |
| 3 | 176 |
| 4 | S2Lung |
| 5 | 106 |
| 6 | 105 |
| 7 | 171/25/02 |
| 8 | C24S11 |
| 9 | AP2 |
| 10 | 53 |
| 11 | 109 |
| 12 | S2Ecoli |
| 13 | C10S19 |
| 14 | C28S1 |
| 15 | 19 |
| 16 | 2 |
| 17 | APEC |
| 18 | S29C12 |
| 19 | 23 |
| 20 | 142 |
| 21 | 20 |
| 22 | S2EC |
| 23 | AP3 |
| 24 | C29SIN |
| 25 | 26 |
| 26 | C24S1b |
| 27 | C14S29 |
| 28 | C24S3b |
| 29 | S44C24 |
| 30 | 78/28/02 |
| 31 | C9S3 |
| 32 | S19C25 |
| 33 | Ecoli |
| 34 | C29 |
| 35 | S2Ecoli2 |
| 36 | 3PC |
| 37 | S3C28 |
| 38 | S3HEC |
| 39 | C24S3b(2) |
| 40 | 8 |
| 41 | C13S1b |
| 42 | 28/179/02 |
| 43 | AP3 |
| 44 | 171/25/02 |
| 45 | AP2 |
| 46 | 53 |
| 47 | C10S19 |
| 48 | 19 |
| 49 | 2 |
| 50 | APEC |
| 51 | S29C12 |
| 52 | 23 |
| 53 | AP3 |
| 54 | 142 |
| 55 | 20 |
| 56 | S2EC |
| 57 | C7S14 |
| 58 | 8 |
| 59 | 137/28/02 |
| 60 | S29S23 |
| 61 | C8S1 |
| 62 | 78/28/02 |
