# Supplementary material for: Bacteriophage activity against and characterisation of avian pathogenic Escherichia coli isolated from colibacillosis cases in Uganda
Source: PLoS One. 2020 Dec 15;15(12):e0239107. doi: 10.1371/journal.pone.0239107 (PMC7737885; doi:10.1371/journal.pone.0239107)
Supplement: S2 Results — (DOCX) [file pone.0239107.s007.docx]

**Phylogenetic results of APEC**

| **S/No** | **Sample ID** | **TSPE4.C2(152bp)** | ***YjaA*(211bp)** | ***ChuA*(279bp)** | **PHYLOGENETIC GROUP** |
| --- | --- | --- | --- | --- | --- |
| 1 | AP3 | 0 | 0 | 1 | D |
| 2 | S1Lung | 1 | 0 | 0 | B1 |
| 3 | 176 | 1 | 0 | 0 | B1 |
| 4 | 106 | 1 | 0 | 1 | D |
| 5 | 105 | 1 | 1 | 0 |  |
| 6 | S2 Lung | 1 | 0 | 0 | B1 |
| 7 | 171/25/02 | 0 | 1 | 0 | A |
| 8 | C24S11 | 1 | 0 | 1 | D |
| 9 | AP2 | 1 | 0 | 0 | B1 |
| 10 | 53 | 1 | 1 | 0 |  |
| 11 | 109 | 1 | 0 | 1 | D |
| 12 | S2Ecoli | 1 | 0 | 1 | D |
| 13 | C28S1 | 1 | 0 | 0 | B1 |
| 14 | S29C12 | 1 | 0 | 1 | D |
| 15 | S2EC | 1 | 0 | 1 | D |
| 16 | C7S14 | 1 | 0 | 1 | D |
| 17 | 137/28/02 | 0 | 1 | 0 | A |
| 18 | C8S3 | 1 | 0 | 1 | D |
| 19 | 26 | 0 | 1 | 0 | A |
| 20 | C24S29 | 1 | 0 | 1 | D |
| 21 | EcoliS3 | 1 | 0 | 1 | D |
| 22 | C24S1b | 1 | 0 | 1 | D |
| 23 | C14S29 | 0 | 0 | 1 | D |
| 24 | S2Ecoli2 | 1 | 0 | 1 | D |
| 25 | C8S19 | 1 | 1 | 1 | B2 |
| 26 | C9S19 | 1 | 0 | 1 | D |
| 27 | S3HEC | 1 | 0 | 1 | D |
| 28 | 19-1330 | 1 | 0 | 1 | D |
| 29 | 19-10952 | 1 | 0 | 1 | D |
| 30 | S14 | 1 | 0 | 1 | D |
| 31 | 19 | 1 | 0 | 1 | D |
| 32 | 2 | 1 | 0 | 0 | B1 |
| 33 | 23 | 0 | 0 | 1 | D |
| 34 | 20 | 1 | 0 | 0 | B1 |
| 35 | 82 | 1 | 1 | 0 |  |
| 36 | S3Lug | 1 | 0 | 0 | B1 |
| 37 | 19-10951 | 1 | 0 | 1 | D |
| 38 | C29SIN | 0 | 0 | 0 | A |
| 39 | S29S23 | 0 | 0 | 0 | A |
| 40 | C24S3b | 0 | 0 | 0 | A |
| 41 | S44C24 | 0 | 0 | 0 | A |
| 42 | 78/28/02 | 0 | 0 | 0 | A |
| 43 | C9S3 | 0 | 0 | 0 | A |
| 44 | S19C25 | 0 | 0 | 0 | A |
| 45 | C29 | 0 | 0 | 0 | A |
| 46 | S3C28 | 0 | 0 | 0 | A |
| 47 | Ecoli | 0 | 0 | 0 | A |
| 48 | 3PC | 0 | 0 | 0 | A |
| 49 | C24S3b(2) | 0 | 0 | 0 | A |
| 50 | 8 | 0 | 0 | 0 | A |
| 51 | C13S1b | 0 | 0 | 0 | A |
| 52 | 28/179/02 | 0 | 0 | 0 | A |
| 53 | C10S19 | 0 | 0 | 0 | A |
| 54 | APEC | 0 | 0 | 0 | A |
| 55 | 142 | 0 | 0 | 0 | A |
| 56 | C8S1 | 0 | 0 | 0 | A |
| 57 | 19-1331 | 0 | 0 | 0 | A |
| 58 | 135 | 0 | 0 | 0 | A |

1=Present, 0=Absent

**Agarose gels for phylogenetic analysis of APEC**

| **Gene** | **Band size** |
| --- | --- |
| TSPE4.C2 | 152bp |
| *YjaA* | 211bp |
| *ChuA* | 279bp |

Samples (1P-5P) 10.7.2019


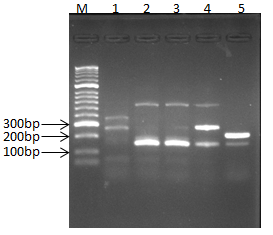


Samples (3P1-3P10) 23.7.2019


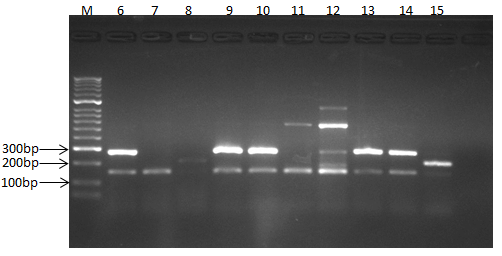


Samples (3P11-3P20)23.7.2019


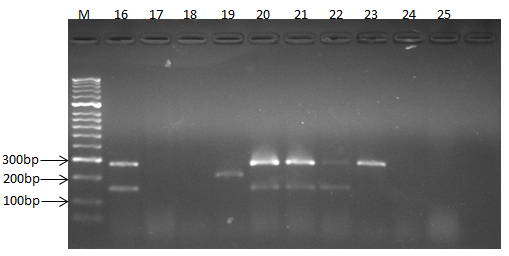


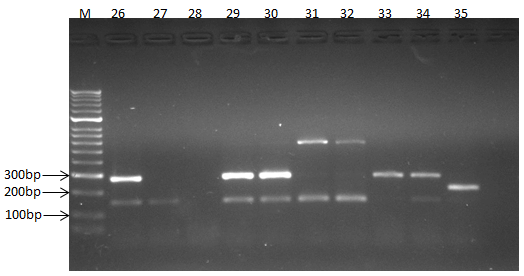
Samples (4P1-4P10) 25.7.2019

Samples (5P1-5P10)29.7.2019


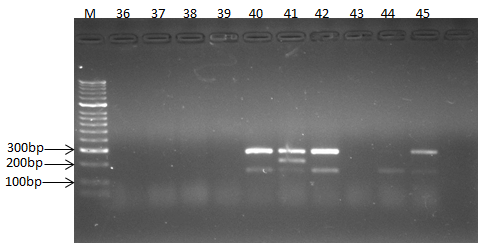


Samples (5P11-5P20) 29.7.2019


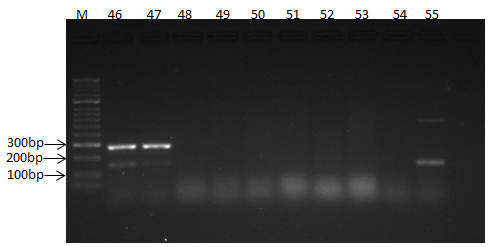


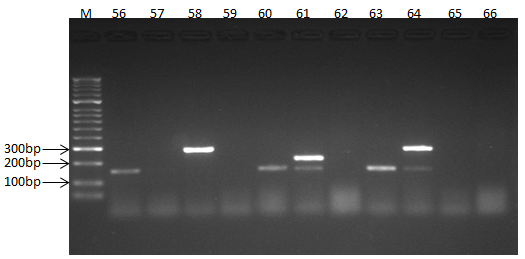
Samples (6P1-6P11) 31.7.2019

| **Lane Number** | **Sample ID** |
| --- | --- |
| M | Marker |
| 1 | AP3 |
| 2 | S1Lung |
| 3 | 176 |
| 4 | 106 |
| 5 | 105 |
| 6 | C24S11 |
| 7 | AP2 |
| 8 | 53 |
| 9 | 109 |
| 10 | S2Ecoli |
| 11 | C28S1 |
| 12 | S29C12 |
| 13 | S2EC |
| 14 | C7S14 |
| 15 | 137/28/02 |
| 16 | C8S3 |
| 17 | C29SIN |
| 18 | S29S23 |
| 19 | 26 |
| 20 | C24S29 |
| 21 | EcoliS3 |
| 22 | C24S1b |
| 23 | C14S29 |
| 24 | C24S3b |
| 25 | S44C24 |
| 26 | C24S11 |
| 27 | AP2 |
| 28 | 53 |
| 29 | 109 |
| 30 | S2Ecoli |
| 31 | C28S1 |
| 32 | S29C12 |
| 33 | S2EC |
| 34 | C7S14 |
| 35 | 137/28/02 |
| 36 | 78/28/02 |
| 37 | C9S3 |
| 38 | S19C25 |
| 39 | C29 |
| 40 | S2Ecoli2 |
| 41 | C8S19 |
| 42 | C9S19 |
| 43 | S3C28 |
| 44 | S3HEC |
| 45 | 19-1330 |
| 46 | 19-10952 |
| 47 | S14 |
| 48 | Ecoli |
| 49 | 3PC |
| 50 | C24S3b(2) |
| 51 | 8 |
| 52 | C13S1b |
| 53 | 28/179/02 |
| 54 | C10S19 |
| 55 | 19 |
| 56 | 2 |
| 57 | APEC |
| 58 | 23 |
| 59 | 142 |
| 60 | 20 |
| 61 | 82 |
| 62 | C8S1 |
| 63 | S3Lug |
| 64 | 19-10951 |
| 65 | 19-1331 |
| 66 | 135 |
